# Supplementary material for: Patterns of Intron Gain and Loss in Fungi
Source: PLoS Biol. 2004 Nov 30;2(12):e422. doi: 10.1371/journal.pbio.0020422 (PMC532390; doi:10.1371/journal.pbio.0020422)
Supplement: Table S1 — Also available at http://genes.mit.edu/NielsenEtAl/. (4.3 MB ZIP). [file pbio.0020422.st001.zip › NielsenEtAl/html/148.html]

AN1860.1.NCU00360.1.MG05179.1.FG06738.1


```
 CLUSTAL W (1.82) Multiple Sequence Alignments - Introns Inserted


Sequence 1: NCU00360.1	288 aa
Sequence 2: MG05179.1	285 aa
Sequence 3: FG06738.1	289 aa
Sequence 4: AN1860.1	283 aa
Alignment Length: 302 aa
Number Identitical Residues: 118 aa
Alignment Score (without introns) 6283


MG05179.1 	MSAAKAAAKRLVVCGGNGFLG~SRICKYGVTRGWDVISIRQ0LTSKPQNS~RSGEPHWAS
NCU00360.1	MSS---AAKKLIVAGGNGFLG~SRICQAAVHRGWDVTSI--~-------S2RSGQPNWSS
FG06738.1 	MSA----SKKLVVCGGTGFLG~SRICKYAVARGWDVTSI--~-------S2RSGDPRWDT
AN1860.1  	MAS-----KKLVVAGGNGFLG1SRICKSAVARGWSVTSI--~-------S2RSGEPRWDV
          	*::     *:*:*.**.**** ****: .* ***.* **          * ***:*.*  

MG05179.1 	VGPTASPPPWAHQVSWERADIFQPEAWAPLVKGADYVVHSMGILLEADYKGAISGRENPV
NCU00360.1	VSASPSAPSWSTAVSWERADIFRPAEWIALLNGADYVVHSLGILLEADYKGVISGRESPI
FG06738.1 	ISASPSPPPWAHKVSWERGDILRPATYAPLLNGADFVVHSMGILLEADYKGVLSGKESPV
AN1860.1  	VTSSPQRPGWAKSVEWAKADILKPNTYKPFLKDANAVVHSMGILLEADYKGVVQGREPII
          	: .:.. * *:  *.* :.**::*  : .:::.*: ****:**********.:.*:*  :

MG05179.1 	QGLKKAFAAK-----------------PPSDPRQLTYEMMNRDSAITLA~REASKANVGA
NCU00360.1	AGLQKAFSPRHTP--NPLQRRPDDELRPPTNANQLTYEMMNRDSAILLA~KEAANKGVKG
FG06738.1 	AGLQKAFAPIRDRGVDPLAKGQGEDINPPNPKDQFTYEIMNRDSAVTLA~KHAVAAKVNS
AN1860.1  	SGLQKAFSPSKRGSQNPLQRQEGEALKAQEKDGQFTYELMNRDS----A1QESLNEHAST
          	 **:***:.    . ..     ..   .     *:***:*****    * :.:    .  

MG05179.1 	FAYISAAGNAPVLPARYLSTKREAESTIASEFPRMRSVFIRAPFLYDTSRLFTVPMAALT
NCU00360.1	FGYISAAGGAPVLPSRYISTKREAEDVIAREFPDMRSVFFRPPFMYDSSRPVTMPLAAMT
FG06738.1 	FCYISAAAGAPVLPQRYITTKREAEITIANKFPELRSLFVRPSFLFDSSRPMTMPLAAMV
AN1860.1  	FLYISASSGAPILPSRYITTKREAETTIATQLPELRSIFVRPPFMYDSSRKLTLPIALGG
          	* ****:..**:** **::****** .** ::* :**:*.*..*::*:** .*:*:*   

MG05179.1 	GLGTLFNRTTGGVFGNFLGAAAIKPLKAEDVAAAVIEALSEEDVRGPIEVAEIEELANKG
NCU00360.1	MAGSIFNGATGGVLSDFLGSAGTKPLKADLVAEAVVEGLDDTAVKGAVEVPELETLANKG
FG06738.1 	GVGTAFNGLTGNYFKNFIGAAGVKPLKVEAVAEAVVEALGDESVKGAIEVPQIEELASKA
AN1860.1  	FVASQLNALVGNRLN-FFGSMVDKPFQVDMVGEAVVEALEDPSVSGPVGPKKIEALATKA
          	  .: :*  .*. :  *:*:   **::.: *. **:*.* :  * *.:   ::* **.*.

MG05179.1 	WRKGML
NCU00360.1	WRRTML
FG06738.1 	WRKTML
AN1860.1  	WRKSML
          	**: **
```
